# Multiple Cayley-Klein metric learning

**DOI:** 10.1371/journal.pone.0184865

**Published:** 2017-09-21

**Authors:** Yanhong Bi, Bin Fan, Fuchao Wu

**Affiliations:** 1 National Laboratory of Pattern Recognition, Institute of Automation, Chinese Academy of Sciences, Beijing, P.R.China; 2 University of Chinese Academy of Sciences, Beijing, P.R.China; National University of Defense Technology, CHINA

## Abstract

As a specific kind of non-Euclidean metric lies in projective space, Cayley-Klein metric has been recently introduced in metric learning to deal with the complex data distributions in computer vision tasks. In this paper, we extend the original Cayley-Klein metric to the multiple Cayley-Klein metric, which is defined as a linear combination of several Cayley-Klein metrics. Since Cayley-Klein is a kind of non-linear metric, its combination could model the data space better, thus lead to an improved performance. We show how to learn a multiple Cayley-Klein metric by iterative optimization over single Cayley-Klein metric and their combination coefficients under the objective to maximize the performance on separating inter-class instances and gathering intra-class instances. Our experiments on several benchmarks are quite encouraging.

## Introduction

An effective distance metric is of great importance for many computer vision and pattern recognition applications such as clustering [1], retrieval [2, 3] and classification [4, 5]. Researches have shown that the widely used Euclidean metric mainly performs well under isotropic assumption of the data space. Therefore, its performance is usually limited since it can not reasonably reflect the underlying relationships between input instances [6–9]. To take the correlation among different data dimensions into consideration, using Mahalanobis metric is a popular solution.

Due to the difficulty in designing a specific Mahalanobis metric for a specific task, learning a Mahalanobis-like distance metric from labeled data attracts a growing attention over the last years [10, 11]. The underlying idea of Mahalanobis metric learning is to define an application dependent metric which could capture the characteristics of the data. It aims to learn a positive semi-definite (PSD) matrix to define a specific Mahalanobis metric, i.e., *d*^2^(**x**, **y**) = (**x** − **y**)^*T*^ **M**(**x** − **y**). Different learning objectives have been proposed in the literature, for example, to maximize the distances between dissimilar samples and simultaneously constrain the distances between similar samples [12], or to maximize the margin between similar pairs and dissimilar pairs [11].

Although Mahalanobis metric learning has been successfully applied in many applications, it is actually a linear metric. However, it is widely believed that the high dimensional data space encountered in computer vision applications is essentially non-linear. Therefore, researchers have resorted to more complicated non-linear metrics to pursue a higher performance. These attempts include local metric learning [11, 13–16], kernel metric learning [17] and the most recently proposed Cayley-Klein metric learning [18], etc.

This paper follows the work of Cayley-Klein metric learning in [18]. A multiple Cayley-Klein metric learning method is proposed. It effectively learns several Cayley-Klein metrics and their linear combination weights to form a powerful non-linear metric. Each of the combined Cayley-Klein metric is focused on a part of the data space and can be considered as a locally optimized metric on a part of the training data. To achieve this goal, we first partition the training data into different clusters according to their label information. Each cluster is assigned with a local Cayley-Klein metric, whose learning optimization is conducted only on the training data from the related cluster. Once these Cayley-Klein metrics have been learned, their combination weights are optimized by maximizing the distances between inter-class instances and simultaneously restricting the distances between intra-class instances smaller than an upper bound. By combining these local metrics together, it effectively leads to a more powerful and global metric for the whole data space. The local Cayley-Klein metrics and their weights are iteratively optimized towards a high classification performance distance metric.

## Related work

In this section, we will first review some related work under the topic of metric learning. Then, we move to a brief introduction to the Cayley-Klein geometries as a basis of our method.

### Metric learning

When the general Euclidean distance can not fulfill the requirement of many computer vision applications, it is straight-forward to explore the label information and the intrinsic structure of training data to learn a specific but more powerful distance metric for a given task.

Most works in the literature have been focused on the Mahalanobis metric learning. The earlier work for Mahalanobis metric learning is the MMC proposed by Xing et al. [12]. It aims to learn a positive semi-definite metric matrix by maximizing the distances between instances from different classes while restricting the distances between instances from a same class smaller than a fixed upper bound. Based on this objective, they finally formulated the metric learning problem as a convex optimization problem which is solved by semidefinite programming. Similar objective has been used in Davis et al. [10] as constraints. Subject to these constraints, Davis et al. proposed the Information Theory Metric Learning (ITML) by minimizing the differential relative entropy. Instead of restricting the intra-class distances below an upper bound, Globerson and Roweis [19] proposed to make them as zero. Guillaumin et al. [20] proposed a discriminative linear logistic regression for Mahalanobis metric learning. Other famous works include the LMNN [11], which tried to learn a Mahalanobis distance metric so as to make the *k*-nearest neighbors always lie in the same class while instances from different classes are separated by a large margin. By replacing the exponential loss in LMNN with the hinge loss, Shen et al. [21] proposed the BoostMetric. They further proposed the FrobMetric by adding a general Frobenius norm as a regularization term to the objective function [22]. More recently, Lu et al. [23] proposed a neighborhood repulsed metric learning method for kinship verification. Their target is to learn a distance metric so that the intra-class samples are pulled as close as possible and inter-class samples lying in a neighborhood are repulsed and pushed away as far as possible. Wang et al. [24] proposed the Shrinkage Expansion Adaptive Metric Learning (SEAML). Their method could adaptively adjust the bound constraints used in previous works [10, 12] by shrinking the distances between samples of similar pairs and expanding the distances between samples of dissimilar pairs. Law et al. [25] proposed the Fantope regularization and applied it to the Mahalanobis metric learning.

Beyond Mahalanobis metric learning, a lot of researchers have also made a big effort to non-Mahalanobis metric learning due to its potential in dealing with more complex intra- and inter-class variations. Kernel trick is the most straight-forward technique to deal with non-linearity, so it is naturally to use kernel method in metric learning, such as [17, 26]. Non-Euclidean spaces such as Riemannian space, projective space have also been explored for metric learning. These methods include Riemannian and manifold metric learning [27, 28] and Cayley-Klein metric learning [18]. In [27], Cheng proposed the Riemannian similarity learning by tackling the metric learning problem in a Riemannian optimization framework. In [18], Bi et al. shown that Cayley-Klein metric can be incorporated into the metric learning frameworks of MMC [12] and LMNN [11] to obtain a better distance metric. Besides, Li et al. [29] proposed a margin based method to learn a second-order discriminant function as distance metric for verification problem. Some researchers have embedded metric learning into the framework of deep neural networks [30, 31].

Since our method learns several Cayley-Klein metrics locally and combines them together for a global and powerful distance metric, it is mostly related to the local metric learning [11, 13, 15, 32] and some mixed/compositional metric learning methods [16, 33]. MM-LMNN [11] is an extension of LMNN which learns a small number of metrics (typically one per class) in an effort to alleviate overfitting. Noh et al. [32] pointed out that finite sampling using the class conditional probability distribution leads to a theoretical bias of the nearest neighbor classifier. Thus they proposed the Generative Local Metric Learning (GLML) using local metrics to limit this theoretical bias. In [13], Wang et al. introduced a local metric learning method based on finite number of linear metrics named PLML. They used the *k*-means algorithm to define some anchor points as the means of clusters and optimized a combination of metric bases learned from these clusters. Reduced-Rank Local Metric Learning (R^2^LML) proposed in [15] learns *k* Mahalanobis-like local metrics that are then conically combined. Additionally, a nuclear norm regularizer is adopted to obtain low-rank weight matrices for calculating metrics, which is able to control the rank of the involved linear mappings through a sparsity-inducing matrix norm. Recently, Semerci and Alpaydin [16] proposed the Mixture of LMNN (MoLMNN) method to learn a mixture of local Mahalanobis distances to better discriminate the data. It needs a gating function to softly partition the input space into several regions. In [33], SCML-local aims to learn a sparse combination of locally discriminative metrics. This algorithm do not need to perform projections onto the PSD cone, thus getting a computational advantage for high-dimensional problems.

Different from these methods, the proposed multiple Cayley-Klein metric learning linearly combines several local Cayley-Klein metrics while most previous methods combine Mahalanobis metrics. Due to the intrinsic non-linearity of Cayley-Klein metric, combining them is more effective than combining linear metrics like Mahalanobis metrics. Thus, our method is potentially to have a better performance than previous methods. Moreover, contrast to the sophisticated methods in the previous works for partitioning the input data space into several clusters for local metrics learning, we use a simpler and straight-forward method by directly utilizing the label information supplied with the training data.

### Cayley-Klein geometries

Cayley-Klein geometries are branches of non-Euclidean geometry, which is an ancient topic in geometry and can be traced back to the 19th century. Among many mathematicians who conducted research on this topic, there were A. Cayley and F. Klein. In 1859, A. Cayley discovered that Euclidean geometry can be considered as a special case of projective geometry which leads to his famous statement “descriptive geometry (his term for projective geometry) is all geometry” [34]. Ten years later, F. Klein [35, 36] followed A. Cayley’s ideas and showed that the projective geometry can provide a framework for the development of hyperbolic and elliptic geometries as well. His research is mainly focused on the real Euclidean, hyperbolic and elliptic geometries since he believed that only these geometries can describe the physical universe [37]. Based on their researches, it is acknowledged that the Euclidean, the hyperbolic and the elliptic geometries are independent and self-subsistent geometries. Their research also leads to working models for these different geometries. Owing to their distinguished work on this topic, both the hyperbolic and elliptic geometries are called Cayley-Klein geometries. They occupy a significant position in the foundations of geometry, because of their distinguished position as geometries of constant curvature.

Nowadays, the term “non-Euclidean geometry” is frequently used to refer the hyperbolic geometry only [38] or the hyperbolic and elliptic geometries together [39]. The reason of calling them “non-Euclidean” is perhaps that no other non-Euclidean geometry had been discovered earlier, and also for which, they both violate the parallel postulate of Euclidean geometry. In Euclidean geometry, for each tangent to a circle there is a unique second parallel tangent. That is to say, there is a unique line through a fixed point in parallel with a given line (not through the fixed point). Whereas in elliptic geometry, there are no parallels at all. As great circles are taken to be lines in elliptic geometry, two different lines in one plane always intersect. In hyperbolic geometry, through one point not on the given line, there are infinitely many parallels to this line.

## Methods

### Cayley-Klein metric

According to [34, 35], Cayley-Klein metric is defined over an invertible symmetric matrix **G** in projective space. Mathematically, the Cayley-Klein distance between two data points $x_{i},x_{j}\in R^{n}$ in *n*-dimensional space is defined as:

$$\begin{array}{c} d_{CK}\left(x_{i},x_{j};G\right)=\frac{k}{2}|\log(\frac{\sigma_{x_{i}x_{j}}+\sqrt{\sigma_{x_{i}x_{j}}^{2}-\sigma_{x_{i}x_{i}}\cdot \sigma_{x_{j}x_{j}}}}{\sigma_{x_{i}x_{j}}-\sqrt{\sigma_{x_{i}x_{j}}^{2}-\sigma_{x_{i}x_{i}}\cdot \sigma_{x_{j}x_{j}}}})|\left(k>0\right) \end{array}$$ (1)

where

σ(xi,xj)=(xiT,1)G(xj1)≜σxixj (2)

*k* is a parameter related to the space curvature [18].

Apparently, there is one-to-one correspondence between the symmetric matrix $G\in R^{\left(n+1)\times (n+1\right)}$ and the Cayley-Klein metric, i.e., a specific **G** defines a specific kind of Cayley-Klein metric. For this reason, **G** is called the Cayley-Klein metric matrix. Depending on whether **G** is positive definite or indefinite, there exist two kinds of Cayley-Klein metric. When **G** is positive definite, *d*_*CK*_ is an elliptic Cayley-Klein metric. Otherwise, *d*_*CK*_ is a hyperbolic Cayley-Klein metric. Bi et al. [18] have shown that a special form of Cayley-Klein metric could approach Mahalanobis metric in an extreme case. For this reason, they also call it the generalized Mahalanobis metric. In their work, two specific metric learning methods have been proposed for learning data-dependent Cayley-Klein metric matrix.

### Multiple Cayley-Klein metrics

In many computer vision tasks, it is expected that data points from same class are localized near each other in the feature space, while data points from different classes are far from each other. On one hand, a distance metric learned for one class may not perform well when applying to another class. On the other hand, a single distance metric learned on data from all classes is usually incompetent to model the multiclass decision boundaries due to the complexity of high dimensional data space. Based on these reasons, we propose the multiple Cayley-Klein metric. It combines multiple Cayley-Klein metrics that are trained on different parts of the training set. Since Cayley-Klein metric is a kind of non-linear metric, combining several metrics could enlarge its non-linearity, thus leading to a better performance.

The definition of multiple Cayley-Klein metric is simple,

$$\begin{array}{c} d_{mCK}\left(x_{i},x_{j}\right)=\sum_{c=1}^{N}\alpha_{c}d_{CK}\left(x_{i},x_{j};G_{c}\right)(\alpha_{c}>0,\sum_{c}\alpha_{c}=1) \end{array}$$ (3)

Essentially, it linearly combines *N* different Cayley-Klein metrics, so it fulfills the metric axioms as well. Note that *d*_*CK*_(**x**_*i*_, **x**_*j*_; **G**_*c*_) is a Cayley-Klein metric learned on the *c*-th data cluster. When the label information is available in the training data, we cluster training data by their labels. In other words, *d*_*CK*_(**x**_*i*_, **x**_*j*_; **G**_*c*_) is learned to maximize the performance related to the *c*-th class. For example, making the distance between any two instances in the *c*-th class small and the distance between instance in the *c*-th class and instance from other classes large. In this case, *N* is set equal to the number of classes. If the label information is unavailable, the training data can be partitioned into *N* clusters by any unsupervised clustering method, such as *k*-means. In this paper, we only focus on the supervised case as the purpose of metric learning is to leverage metric’s performance by using labeled training data.

**Fig 1:**
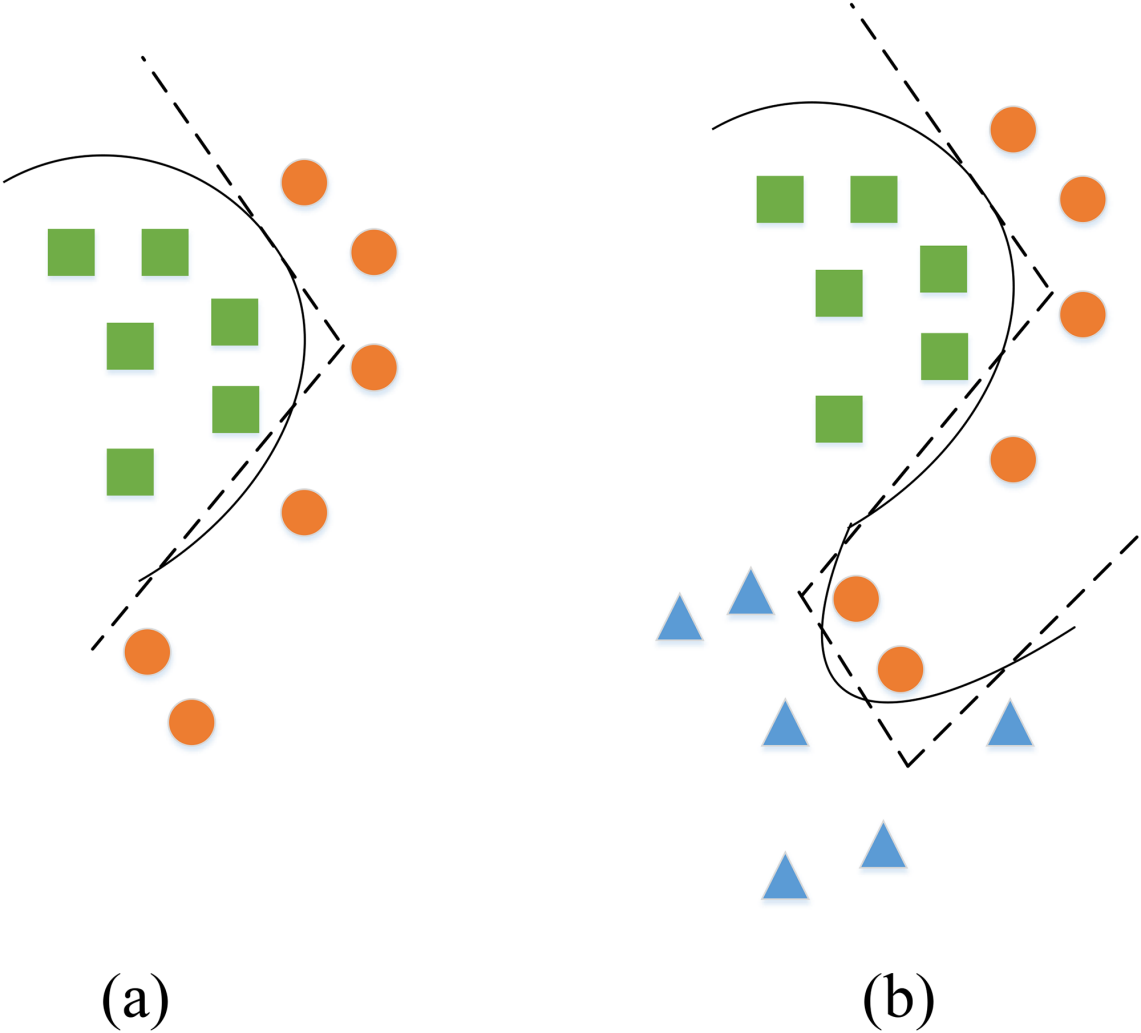
Intuitive illustration of multiple Cayley-Klein metrics and single Cayley-Klein metric by a toy example. (a) non-linear metric VS. linear metrics. (b) multiple non-linear metrics VS. single non-linear metric.

Fig 1 illustrates the basic idea of the proposed multiple Cayley-Klein metric learning method by a toy example. There are two classes of data in (a) denoted by squares and circles, three classes of data in (b) denoted by squares, circles and triangles respectively. In situation (a), we can see that using a non-linear metric achieves the same goal as using two linear metrics in data classification. While in situation (b), a single non-linear metric is not enough, it would need at least two non-linear metrics or even more linear ones to separate the data. Therefore, multiple Cayley-Klein metrics actually correspond to a series of Riemannian metrics with several different (but fixed) curves, which we expect to model more complex data distribution.

In the following, we will describe the formulation of multiple Cayley-Klein metric learning, and then elaborate how to optimize the objective function.

### Metric learning

Suppose we have a training set of *N* classes. According to the label information, we organize it into *N* sets of similar pairs $S=\{S_{c},c=1,2,\cdots ,N\}$ and *N* sets of dissimilar pairs $D=\{D_{c},c=1,2,\cdots ,N\}$. In $S_{c}$, it is constituted by samples from the *c*-th class. While in $D_{c}$, it contains pairs of dissimilar samples, one of which from the *c*-th class, and the other from the *j*-th class, *j* ≠ *c*. Following the widely used learning criteria in metric learning community, we formulate our objective as follows:

$$\begin{array}{c} \begin{array}{cc} \underset{\alpha_{c},G_{c},c=1,2,\cdots ,N}{maximize} & \sum_{\left(x_{i},x_{j}\right)\in D}d_{mCK}\left(x_{i},x_{j}\right)\\ subject\;to & \left(a\right)\;\sum_{\left(x_{i},x_{j}\right)\in S}d_{mCK}\left(x_{i},x_{j}\right)\leq 1\\ & \left(b\right)\;0\leq \alpha_{c}\leq 1,\;\sum_{c=1}^{N}\alpha_{c}=1\\ & \left(c\right)\;G_{c}>0 \end{array} \end{array}$$ (4)

Our objective is to learn a multiple Cayley-Klein metric such that the distances of dissimilar pairs as max as possible, while in the meantime restricting the distances of similar pairs to be smaller than 1. Directly optimize the above problem is difficult. Here, we propose to optimize *α*_*c*_ and **G**_*c*_ alternatively.

***Optimize α***. Given *N* Cayley-Klein matrices **G**_*c*_, the problem to solve *α*_*c*_ is formulated as:

$$\begin{array}{ll} \underset{\alpha_{c},c=1,2,\cdots ,N}{maximize} & \sum_{c=1}^{N}\alpha_{c}\left(\sum_{\left(x_{i},x_{j}\right)\in D^{\prime }}d_{CK}\left(x_{i},x_{j};G_{c}\right)\right)\\ subject\;to & \left(a\right)\;\sum_{c=1}^{N}\alpha_{c}\left(\sum_{\left(x_{i},x_{j}\right)\in S}d_{CK}\left(x_{i},x_{j};G_{c}\right)\right)\leq 1\\ & \left(b\right)\;0\leq \alpha_{c}\leq 1,\;\sum_{c=1}^{N}\alpha_{c}=1 \end{array}$$ (5)

Such a linear programming problem is easy to solve. Note that concatenating all sets of dissimilar pairs $D_{c},c=1,2,\cdots ,N$ contains duplicated pairs. $D^{\prime }$ is the set of dissimilar pairs after removing duplicated pairs from D.

***Optimize **G**_*c*_***. Once the weights are fixed, the problem in (4) could be separated into *N* sub-problems, which are solved one by one. For the *c*-th sub-problem, it is:

$$\begin{array}{ll} \underset{G_{c}}{maximize} & \sum_{\left(x_{i},x_{j}\right)\in D_{c}}\alpha_{c}d_{CK}\left(x_{i},x_{j};G_{c}\right)\\ subject\;to & \left(a\right)\;\alpha_{c}\sum_{\left(x_{i},x_{j}\right)\in S_{c}}d_{CK}\left(x_{i},x_{j};G_{c}\right)\\ & \;+\sum_{p\neq c}\alpha_{p}\sum_{\left(x_{i},x_{j}\right)\in S_{p}}d_{CK}\left(x_{i},x_{j};G_{p}\right)\leq 1\\ & \left(b\right)\;G_{c}>0 \end{array}$$ (6)

Since matrix **G**_*c*_ in the objective is symmetric, it is convenient to optimize on **L**_*c*_ after Cholesky decomposition $G_{c}=L_{c}^{T}L_{c}$ with $L_{c}\in R^{\left(n+1)\times (n+1\right)}$. In this way, the above problem can be solved by the gradient ascend algorithm. At each iteration, we take a gradient ascent step on the objective function

$$\begin{array}{c} \varepsilon \left(L_{c}\right)=\sum_{\left(x_{i},x_{j}\right)\in D_{c}}\alpha_{c}d_{CK}\left(x_{i},x_{j};L_{c}\right) \end{array}$$ (7)

with respect to **L**_*c*_. By applying the Cholesky decomposition on **G**_*c*_, constraint (b) is satisfied. Then we just need to approximate the updated **L**_*c*_ to fulfill the constraint (a). Specifically, given an updated **L**_*c*_, its approximated **L**′ that meets the constraints (a) can be obtained by the following minimization problemml:

$$\begin{array}{ll} minimize_{L^{\prime }} & ∥L^{\prime }-L_{c}{∥}_{F}\\ subject\;to & \alpha_{c}\sum_{\left(x_{i},x_{j}\right)\in S_{c}}d_{CK}\left(x_{i},x_{j};L^{\prime }\right)\\ & \;+\underset{p\neq c}{\sum }\alpha_{p}\sum_{\left(x_{i},x_{j}\right)\in S_{p}}d_{CK}\left(x_{i},x_{j};L_{p}\right)\leq 1 \end{array}$$ (8)

For simplicity, we denote $C_{x_{i}x_{j}}={\left(x_{i}^{T},1\right)}^{T}\left(x_{j}^{T},1\right)$, then:

$$\begin{array}{c} \sigma \left(x_{i},x_{j}\right)=tr\left(C_{x_{i}x_{j}}G_{c}\right)=tr\left(C_{x_{i}x_{j}}\left(L_{c}^{T}L_{c}\right)\right) \end{array}$$ (9)

Suppose the matrix **L**_*c*_ at the *t*-th iteration is **L**^*t*^, we can compute the gradient of the objective function at the *t*-th iteration as:

$$\begin{array}{c} \begin{array}{cc} L^{t} & =\frac{\varepsilon (L_{c})}{\partial L_{c}}{|}_{L^{t}}=\frac{k}{2i}\cdot 2L_{c}\cdot \alpha_{c}\cdot \sum_{\left(x_{i},x_{j}\right)\in D_{c}}(\frac{2C_{ij}}{\sqrt{\sigma_{ij}^{2}-\sigma_{ii}\sigma_{jj}}}\\ & \;\;\;\;-\frac{\sigma_{ij}C_{ii}}{\sigma_{ii}\sqrt{\sigma_{ij}^{2}-\sigma_{ii}\sigma_{jj}}}-\frac{\sigma_{ij}C_{jj}}{\sigma_{jj}\sqrt{\sigma_{ij}^{2}-\sigma_{ii}\sigma_{jj}}}) \end{array} \end{array}$$ (10)

***Initialization***. To start the alternative optimization procedure described above, we have to initialize *α*_*c*_ and **G**_*c*_ in a reasonable way. Bi et al. [18] have proposed a specific method to construct a Cayley-Klein matrix from a given dataset, which is called the generalized Mahalanobis matrix. They have experimentally shown a better performance of initialization using the generalized Mahalanobis matrix compared to using an identity matrix or a random matrix. Therefore, we also choose to use the generalized Mahalanobis matrix to initialize **G**_*c*_. Since **G**_*c*_ is a local metric mainly focused on the *c*-th class, we use the mean **m**^(*c*)^ and inverse covariance *Σ*^(*c*)^ computed from samples of the *c*-th class. In this way, we initialize **G**_*c*_ with the following matrix:

$$\begin{array}{c} G_{c}=(\begin{array}{cc} \Sigma^{\left(c\right)} & -\Sigma^{\left(c\right)}m^{\left(c\right)}\\ -m^{(c)T}\Sigma^{\left(c\right)} & m^{(c)T}\Sigma^{\left(c\right)}m^{\left(c\right)}+k^{(c)2} \end{array})\;\left(k^{\left(c\right)}>0\right) \end{array}$$ (11)

For *α*_*c*_, it is simply initialized as 1/*N*.

Combining all the above issues together, we summarize the proposed Multiple Cayley-Klein Metric Learning (MCKML) algorithm as follows:

**Algorithm 1**. Multiple Cayley-Klein Metric Learning (MCKML)

**Input**: classes of labeled training data (organized into sets of similar pairs and sets of dissimilar pairs), convergence error *ϵ*.

**Output**: *α*_*c*_,**G**_*c*_, *c* = 1, 2, ⋯, *N*

**Begin**

1. Set *α* = 1/*N* and **G**_*c*_ according to Eq (11)
2. Optimize *α* by solving (5) with linear programming.
3. **for** *c* = 1 to *N* **do**
4.  Optimize **G**_*c*_ by solving (6).
5. **end for**
6. Repeat 2–5 until $\sum_{c=1}^{N}\left|G_{c}^{update}-G_{c}^{previous}\right|<\epsilon$, where $G_{c}^{update}$ and $G_{c}^{previous}$ denote the updated and the previous **G**_*c*_ respectively.
7. **return** *α* and **G**_*c*_

**End**

## Experiments

In this section, we evaluate the proposed method on image classification tasks with three different public datasets. For comparison, we also tested the performance of CK-MMC and MMC as they share an identical learning target as our method. Their difference only lies in the definition of distance metric. Moreover, LMNN and CK-LMNN have been evaluated due to their good performance. Additionally, MM-LMNN and SCML-local also have been tested as they are typical local metric learning methods.

### Results on the UCI datasets

**Datasets**: In this experiment, we use 9 different datasets from the UCI Machine Learning Repository at http://archive.ics.uci.edu/ml/datasets.html, which are widely used in evaluating metric learning methods. These datasets include: Wine, Ionosphere, Vowel, Balance, Pima, Vehicle, Segmentation, Waveform and Letter. The characteristics of each UCI dataset such as the number of data points, feature dimensions, and the number of classes are summarized in Table 1.

**Table 1:** Characteristics and experiment settings of the UCI datasets.

Datasets	Data points	Training	Validation	Test	Attributes	Classes
Wine	178	107	35	36	13	3
Iono.	351	210	70	71	34	2
Vowel	528	317	105	106	10	11
Bala.	625	375	125	125	4	3
Pima	768	461	153	154	8	2
Vehicle	846	507	169	170	18	4
Seg.	2310	1386	462	462	19	7
Wave	5000	3000	1000	1000	21	3
Letter	20000	3000	1000	1000	16	26

**Set up**: For each dataset, we randomly divide it into training/validation/test sets. The numbers of samples in the training/validation/test subsets are shown in Table 1, and the proportion of these three subsets is nearly 60%/20%/20%. All features are first normalized over the training data to have zero mean and unit variance. Features of the validation and test data are normalized using the mean and variance of training data. The parameters of all methods are set by authors’ recommendation. LMNN, MM-LMNN and CK-LMNN use 3 target neighbors and all imposters, while these are set to 3 and 10 in SCML-Local. The *k*-nearest neighbor (*k*NN) classifier is used for classification, and we set *k* = 3 for all the datasets. We repeat this procedure 10 times and report the average accuracies for these datasets.

**Results**: Table 2 shows the classification accuracies for the seven evaluated methods. Consistent to the previous work, the performance is improved by using Cayley-Klein metric to replace the traditional Mahalanobis metric. This point can be read from “CK-MMC VS. MMC” and “CK-LMNN VS. LMNN”. Among all the evaluated methods, the proposed MCKML performs the best on 6 out of 9 datasets. For two datasets (Balance and Letter), it performs the second best and closely follows the best result (SCML-local). Note that CK-LMNN, MM-LMNN and SCML-local use a learning target based on triplets of samples that is more powerful than the learning target based on pairs of samples, which is used in MCKML. When considering the same learning target, MCKML consistently improves over MMC and CK-MMC on all datasets. By incorporating MCKML to the learning paradigm of LMNN, it is expected to further improve its performance. We will leave this as our future work.

**Table 2:** Classification accuracies (mean and standard deviation in %) on UCI datasets. MCKML achieves the best performance on 6 out of 9 datasets.

Method	MMC [12]	LMNN [11]	CKMMC [18]	CKLMNN [18]	MMLMNN [11]	SCML-local [33]	MCKML
Wine	94.8±2.8	96.2±2.3	95.7±2.7	96.8±2.4	96.9±2.3	97.2±2.4	**97.6**±2.4
Iono.	84.5±1.5	86.7±1.4	84.8±1.4	87.2±1.4	88.7±1.4	89.7±1.5	**89.8**±1.4
Vowel	89.4±1.4	95.1±1.3	92.4±1.5	95.5±1.3	95.2±1.1	95.0±1.2	**95.8**±1.3
Bala.	86.0±2.0	87.3±1.7	86.2±1.9	88.7±1.7	89.6±1.8	**92.3**±1.8	90.7±1.7
Pima	68.6±1.9	70.3±1.6	71.9±1.8	71.5±1.6	71.8±1.7	70.3±1.6	**72.9**±1.7
Vehicle	70.1±2.5	75.4±2.3	78.1±2.4	78.0±2.3	78.4±2.4	79.8±2.4	**81.7**±2.3
Seg.	94.6±1.4	96.2±1.2	96.9±1.3	97.0±1.2	97.0±1.2	97.1±1.2	**97.5**±1.3
Wave	80.9±1.1	81.5±0.8	81.0±1.2	82.8±1.0	82.8±1.1	**83.0**±1.2	82.7±1.1
Letter	89.6±1.3	91.2±1.2	90.9±1.3	92.0±1.2	92.3±1.2	**92.5**±1.2	92.3±1.2
avg.	84.3±1.8	86.6±1.5	86.4±1.7	87.7±1.6	88.1±1.6	88.5±1.6	**89.0**±1.6

For more accurate comparison, we perform paired *t*-test with significance level 0.05 to statistically evaluate which result is better. The comparison results with CK-MMC, CK-LMNN and two local metric learning methods (MM-LMNN and SCML-local) are summarized in Table 3. We use “∼”to indicate the classification results of the two methods are not significantly different for the given confidence level, and “<” to indicate that the mean of the classification accuracy of the latter method is statistically higher than that of the former one. From the paired *t*-test results, we can conclude with a 95% confidence level that the proposed MCKML generally outperforms CK-MMC and is comparable with or even better than CK-LMNN, MM-LMNN and SCML-local on all datasets except Balance dataset.

**Table 3:** Paired *t*-test for statistical evaluation of the classification results on UCI datasets.

Datasets	Paired *t*-test
Wine	CKMMC < CKLMNN ∼ MMLMNN < SCML-local ∼ MCKML
Iono.	CKMMC < CKLMNN < MMLMNN < SCML-local ∼ MCKML
Vowel	CKMMC < SCML-local ∼ MMLMNN < CKLMNN ∼ MCKML
Bala.	CKMMC < CKLMNN < MMLMNN < MCKML < SCML-local
Pima	SCML-local < CKLMNN ∼ MMLMNN ∼ CKMMC < MCKML
Vehicle	CKLMNN ∼ CKMMC ∼ MMLMNN < SCML-local < MCKML
Seg.	CKMMC ∼ CKLMNN ∼ MMLMNN ∼ SCML-local < MCKML
Wave	CKMMC < MCKML ∼ CKLMNN ∼ MMLMNN ∼ SCML-local
Letter	CKMMC < CKLMNN < MMLMNN ∼ MCKML ∼ SCML-local

**Fig 2:**
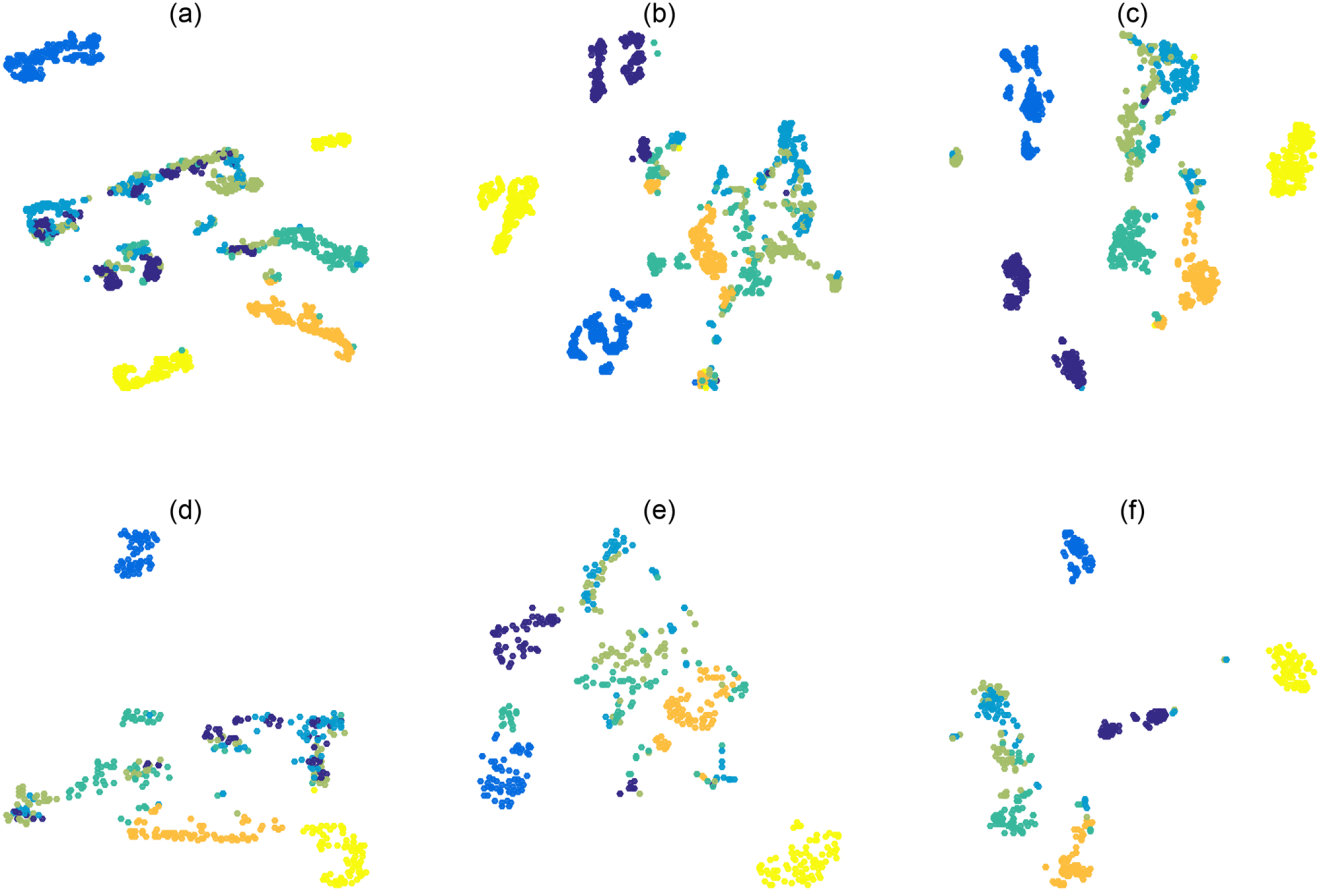
Illustrative experiment on Segmentation dataset in 2D. (a)∼(c) Distributions of training points under the learned metrics (MMC, CK-MMC and MCKML) respectively. (d)∼(f) Distributions of test points under the learned metrics (MMC, CK-MMC and MCKML) respectively.

**Visualization of the learned metric**: In order to provide a better understanding of why the proposed MCKML works well and further show the necessity (benefit) of enlarging non-linear property, we added a graphical illustration using t-SNE [40] on the Segmentation dataset with MMC, CK-MMC and MCKML. In the first row of Fig 2, we can see that although CK-MMC improves MMC, MCKML obtains further improvement. Under the metric obtained by MCKML, the distributions of different classes (denoted by different colors) are more concentrated. Meanwhile, each class is far from other classes and the boundaries are more clear and legible. The second row shows that the metrics consistently generalize to test data. Such a visualization validates the necessity to use the Cayley-Klein metric as well as the multiple Cayley-Klein metric.

### Results on the PubFig dataset

**Dataset**: *Public Figure Face Database (PubFig)* [41] is a challenging real-world face database collected from the internet. It contains 200 people and has a total number of 58,797 images of them. The images in this database are taken in completely uncontrolled situations with non-cooperative subjects, leading to large variations in pose, lighting, expression, scene, camera, imaging conditions and parameters, etc. Similar to [18, 25], our experiment uses a subset of PubFig, containing 772 images from 8 identities, including Alex Rodriguez (**Alex**), Clive Owen (**Clive**), Hugh Laurie (**Hugh**), Jared Leto (**Jared**), Miley Cyrus (**Miley**), Scarlett Johansson (**Scarlett**), Viggo Mortensen (**Viggo**) and Zac Efron (**Zac**). We use 11-dimensional relative attributes [42] to represent each image in the dataset. The relative attributes are computed from a concatenation of the 512-dimensional GIST descriptor [43] and a 45-dimensional LAB color histogram. We use the publicly available codes of [42] to compute relative attributes.

**Set up**: For all the evaluated methods, we randomly select 30 images per class for training, 30 images per class for validation, and use the remaining images for testing. In the test stage, we use a 3-NN classifier based on the learned distance metric. We repeat this procedure 10 times and report the average classification accuracies.

**Results**: The results are listed in Table 4. We could obtain similar observations as in the UCI datasets: MCKML outperforms MMC and CK-MMC in all cases, while it is slightly inferior to CK-LMNN in some categories (the reason has been explained in the last subsection). Moreover, two local metric learning methods MM-LMNN and SCML-local, which all use a set of triplet constraints as LMNN, perform better than LMNN while comparable to CK-LMNN. When comparing the overall performance, MCKML is the best. By comparing the results of MCKML to those of CK-MMC and CK-LMNN, it is clear that learning multiple Cayley-Klein metrics does improve the performance of learning a single Cayley-Klein metric. Although Cayley-Klein metric learning already improves the traditional Mahalanobis metric learning, the multiple Cayley-Klein metric learning further improves its performance.

**Table 4:** Classification accuracies for each identity (mean in %) and average accuracies (mean and standard deviation in %) obtained on the PubFig dataset.

Method	MMC [12]	LMNN [11]	CKMMC [18]	CKLMNN [18]	MMLMNN [11]	SCML-local [33]	MCKML
Alex	79.2	80.1	84.3	81.6	81.0	82.1	**85.7**
Clive	72.7	75.8	83.8	82.5	80.4	83.0	**84.5**
Hugh	83.4	80.6	85.8	83.7	82.7	83.4	**86.4**
Jared	79.2	80.2	79.9	**81.1**	80.6	80.2	80.6
Miley	77.9	75.4	78.2	78.7	77.9	78.9	**81.8**
Scarlett	84.3	83.1	83.0	84.1	84.8	85.2	**86.8**
Viggo	77.3	77.9	78.4	**79.9**	78.4	79.1	79.1
Zac	84.9	82.1	85.2	84.3	83.5	84.9	**88.2**
avg.	79.9±1.2	79.4±1.9	82.3±0.9	82.0±1.9	81.2±1.8	82.1±1.1	**84.1**±1.1

### Results on the OSR dataset

**Dataset**: *Outdoor Scene Recognition Dataset (OSR)* [43] contains 2688 images from 8 outdoor scene categories: tall buildings (**B**), inside city (**IC**), street (**S**), highways (**H**), coast (**C**), open country (**OC**), mountain (**m**) and forest (**F**). We use the 6-dimensional relative attributes generated from 512-dimensional GIST descriptors to represent the images.

**Set up**: As in the experiment on the PubFig dataset, we randomly select 30 images per class for metric learning, 30 images per class for validation, and use the remaining images to test the performance of the learned metric. 3-NN classifier is used for classification. We repeat this procedure 10 times and report the average classification accuracies.

**Results**: The classification results on the OSR dataset are listed in Table 5. Owing to the more powerful learning objective based on triplets of samples, LMNN/CK-LMNN outperforms MMC/CK-MMC respectively in all categories. In average, there is over 2% improvement. Under the same learning objective, we can see that using Cayley-Klein metric (CK-MMC) outperforms using Mahalanobis metric (MMC) by 3%. The performance of Cayley-Klein metric is further improved by the proposed multiple Cayley-Klein metric by additionally 3%. The local metric learning methods MM-LMNN and SCML-local outperform the original LMNN while inferior to CK-LMNN.

**Table 5:** Classification accuracies for each category (mean in %) and average accuracies (mean and standard deviation in %) obtained on the OSR dataset.

Method	MMC [12]	LMNN [11]	CKMMC [18]	CKLMNN [18]	MMLMNN [11]	SCML-local [33]	MCKML
**B**	69.0	72.7	74.1	75.2	74.7	75.2	**76.3**
**IC**	41.2	45.8	46.3	47.4	46.9	46.5	**48.6**
**S**	64.2	69.6	71.9	74.7	74.1	74.4	**75.9**
**H**	74.6	75.3	75.0	**78.2**	75.5	76.8	77.9
**C**	69.1	70.9	70.1	**72.3**	71.4	71.1	71.9
**OC**	56.3	57.3	57.9	58.8	58.3	59.5	**61.4**
**m**	60.2	62.5	64.0	65.0	64.3	63.9	**68.4**
**F**	85.4	86.3	87.3	88.3	88.2	87.8	**88.9**
avg.	65.0±1.2	67.5±1.1	68.3±1.2	70.0±1.1	69.2±1.1	69.4±1.0	**71.2**±1.0

Finally, we can find that the results in Tables 2, 4 and 5 are rather consistent, although these datasets are fundamentally different from each other. Among all the tested methods, the proposed MCKML achieves the best average classification and only slightly inferior to CK-LMNN which uses a more powerful learning objective based on triplets. When using the same objective based on pairs of samples, our method outperforms previous methods on all tested categories.

Table 6 shows the running times on OSR and PubFig for different methods, which are average results of 10 runs. Generally speaking, using Cayley-Klein metric requires a litter more time in testing as more operations are involved in computing Cayley-Klein metric according to its definition. While for training, compared with MMC and CK-MMC, which all need one loop of gradient ascending to find the optimal solution, MCKML needs two loops that is time consuming. One is the outer loop optimized alternatively on *α* and the Cayley-Klein matrices **G**_*c*_, while the other is the inner loop for solving **G**_*c*_ by gradient ascending identical to CK-MMC. When compared with the other two local metric learning methods, MM-LMNN and SCML-local are more efficient than MCKML.

**Table 6:** Running times on OSR and PubFig.

Method	Training time		Testing time	
	OSR	PubFig	OSR	PubFig
MMC [12]	4.6s	5.8s	0.2s	0.2s
CK-MMC [18]	5.3s	3.3s	0.3s	0.3s
LMNN [11]	3.3s	9.2s	0.3s	0.2s
CK-LMNN [18]	2.7s	4.5s	0.4s	0.3s
MM-LMNN [11]	6.5s	14.7s	0.3s	0.3s
SCML-local [33]	17.7s	13.1s	0.2s	0.2s
MCKML	59.4s	77.3s	0.4s	0.3s

## Conclusion

This paper follows a very recent work of Cayley-Klein metric learning, which is a first paper introducing the ancient Cayley-Klein geometries in computer vision. We show in this paper that Cayley-Klein metric can benefit from learning multiple local Cayley-Klein metrics, each of which is only focused on a part of the data space. To this end, we propose the multiple Cayley-Klein metric learning method, which alternatively optimizes over the local Cayley-Klein metrics and their global combination weights. Although the metric learning target is identical to some previous works, i.e., to maximize the inter-class distances and restrict the intra-class distances to be less than an upper bound, our method results in a better performance on three widely used datasets as shown in the experiments. These results demonstrate the superiority of multiple Cayley-Klein metric learning to the Cayley-Klein metric learning, as well as the traditional Mahalanobis metric learning and the state-of-art local metric learning.
